# Robin Weiss, HIV-1 and CD4

**DOI:** 10.3390/v18101168

**Published:** 2026-10-21

**Authors:** Quentin Sattentau

**Affiliations:** 1 The Sir William Dunn School of Pathology, The University of Oxford, Oxford OX1 3RE, UK; quentin.sattentau@path.ox.ac.uk or quentinjames.sattentau@mdc-berlin.de; 2 The Max Delbrück Centre for Molecular Medicine, Campus Berlin-Buch, 13125 Berlin, Germany

**Keywords:** HIV-1, CD4, viral fusion, neutralising antibodies

## Abstract

Professor Robin Weiss, who died earlier this year, made profound early contributions to how HIV-1 and related immunodeficiency viruses infect cells via the CD4 receptor, and the role of neutralising antibodies in inhibiting HIV-1 infection. These and many other scientific contributions, alongside his mentorship of multiple scientists over decades, continue to impact today’s research and maintain his memory.

Robin Weiss, who died this year, was an outstanding virologist and cancer biologist who made pivotal contributions to our understanding of HIV-1 biology. Robin was quick off the mark when HIV-1 (then called LAV or HTLV-III) was confirmed as the cause of AIDS in 1983. Combining his two areas of expertise, he took the essential step of cultivating HIV-1 isolates in cancer T cell lines and observing their virus-induced fusion into syncytia (Figure 1a). Additional novelty came in the form of the emerging technology of viral pseudotypes: vesicular stomatitis virus (VSV) particles that had incorporated functional HIV-1 envelope glycoproteins (Env) by passage through HIV-1-infected cells (Figure 1a). These tools allowed Paul Clapham and other members of Robin’s lab to rapidly address several fundamental questions concerning HIV-1 biology: what was the cellular receptor for the virus, how was the receptor used to enter cells, and could HIV-1 infection be neutralised by serum antibodies from HIV-1-infected individuals? In each case, Robin’s lab produced definitive answers that guided the field for decades. Robin and Gus Dalgleish collaborated with Peter Beverley, who had access to a large panel of monoclonal antibodies (mAbs) against the T cell surface. Using these they showed [1], in parallel with David Klatzmann in Paris [2], that mAbs to CD4 but not most other T cell surface molecules inhibited VSV-HIV-1 pseudotype infection and HIV-1-induced syncytium formation (Figure 1a). These findings identified CD4 as the primary HIV-1 receptor [1,3], but with typical foresight Robin entitled the paper “The CD4 (T4) antigen is an essential component of the receptor for the AIDS retrovirus”, thus leaving conceptual space for the existence of coreceptors (see below). Further work revealed that related immunodeficiency-causing retroviruses HIV-2 and simian immunodeficiency virus (SIV) also use CD4 as their primary receptor [4,5]. Myra McClure worked with Robin to interrogate evolutionary conservation of CD4 use by SIV amongst primates, thus inferring the role of CD4 in immunodeficiency virus zoonotic spread [5]. Genetic confirmation of the importance of CD4 came from Robin’s collaboration with Richard Axel’s lab, in which Paul Maddon showed that human CD4 expression in human cells of diverse origin was sufficient to render them susceptible to HIV-1 infection and fusion [6]. Human CD4 expression in mouse cells did not, however, make them permissive to HIV-1 infection and fusion [6], raising a conundrum that took a decade to resolve by identifying the HIV-1 coreceptors CXCR4 and CCR5 [7]. The initial rapid discovery phase following the identification of CD4 as the HIV and SIV primary receptor saw other pivotal findings. The CD4 binding site on the HIV-1 Env outer gp120 subunit was mapped to the CD4 N-terminal domain [8,9], stimulating development of soluble CD4-based therapeutic approaches [9]. Myra and Robin collaborated with Mark Marsh to show that unlike viruses such as influenza, which fuse with cells from within endosomes via a low-pH-induced conformational switch, HIV-1 fuses with T cells at neutral pH [10] (Figure 1b). This was consistent with results from Robin’s collaboration with the Axel lab showing that HIV-1 fuses primarily at the cell surface [11]. Together these insights led to the concept of receptor-mediated activation of HIV-1 fusion: John Moore, then in Robin’s lab, and I (then in the Axel lab), showed that CD4-induced conformational changes in the HIV-1 envelope glycoproteins (Env) prepare Env for subsequent membrane fusion events [12,13,14,15]. Finally, and most impactfully with regard to vaccine development, Robin’s and Richard Tedder’s labs revealed that HIV-1 could be neutralised by antibodies present in the sera of HIV-1-infected individuals [16]; see accompanying article in this issue [17]. Interestingly, HIV-1 neutralisation was relatively weak compared to other retroviruses, and contained responses to highly variable epitopes [18], findings that foreshadowed major challenges in developing an HIV-1 vaccine. Robin contributed substantially to HIV-1 antibody-based vaccine design by chairing a Gates Foundation-funded Collaboration for AIDS Vaccine Discovery consortium, within which Antonio Lazaveccia and Davide Corti isolated some of the first broadly neutralising antibodies (bNAbs) to HIV-1 [19]. These included a novel bNAb specific to the CD4 binding site on gp120, which is a major site of vulnerability on HIV-1 Env [20]. Such bNAbs to various Env epitopes inform HIV-1 antibody-based vaccine design approaches [21], which, after a long gestation period, may now be bearing fruit [22].

What is apparent from this brief summary of Robin’s contributions to HIV-1 biology is that he was a highly innovative and collaborative researcher who made profoundly important discoveries early in the HIV-1 pandemic. But this does not describe the person. He was also generous, kind, and a committed humanist. His papers were, against somewhat of a trend at the time, restrained and realistic in their language and scientific claims. His lectures and seminars were a charismatic mix of knowledge, wisdom and wit. He was an honest and fearlessly outspoken critic of some of the sillier behaviour of research institutions and individuals. Robin’s lab, and his broader influence in virology and tumour biology, promoted generations of scientists too numerous to name here, who continue to make impactful discoveries in the fields of HIV-1 molecular and cellular pathogenesis and vaccine design, and beyond. We will all miss him as a scientist and person.

## Figures and Tables

**Figure 1:**
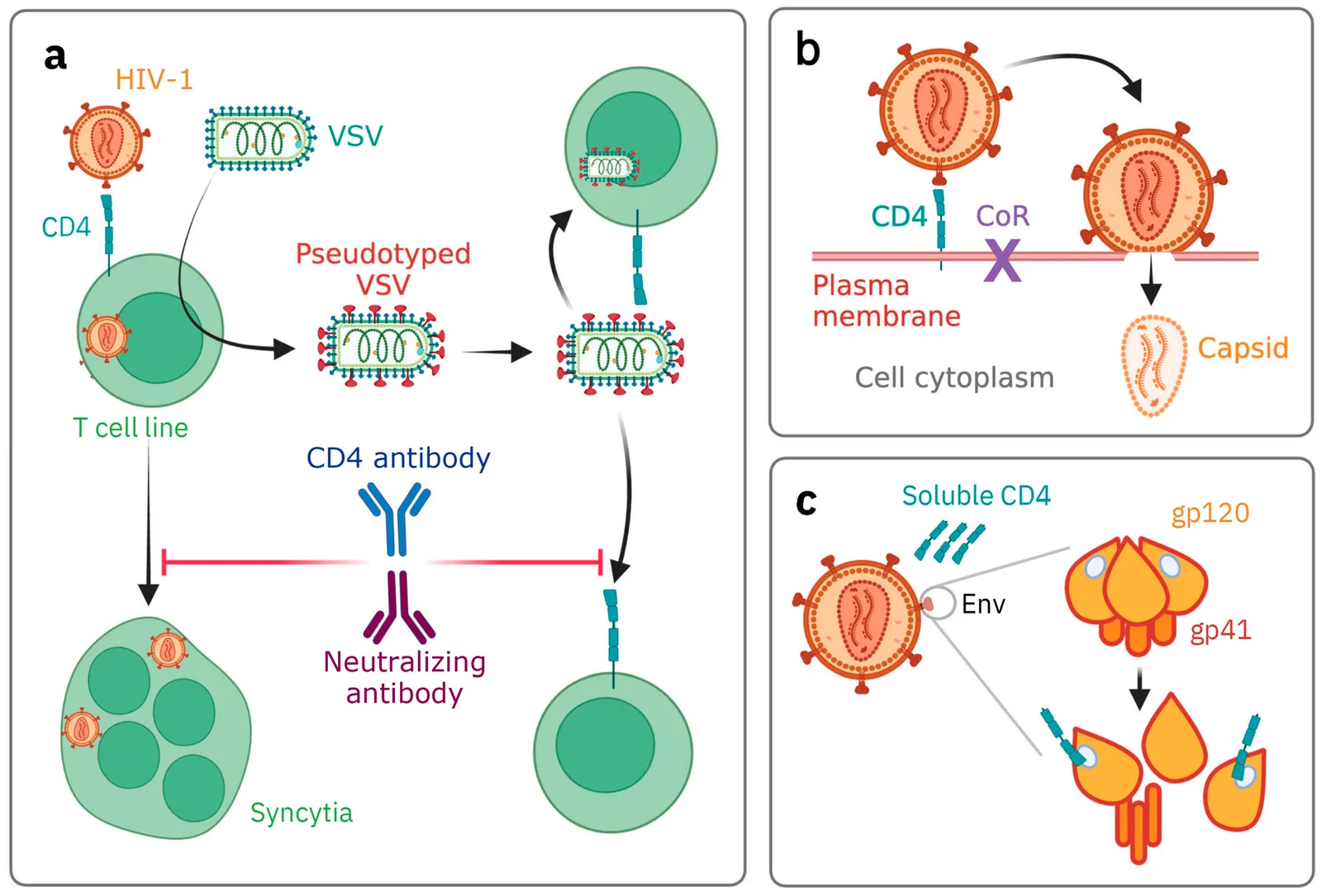
Robin Weiss’s discoveries relating to CD4 and HIV. (**a**) HIV-1 infects CD4-expressing T cell lines, which drive cell–cell fusion and syncytium formation (left). Passage of VSV through HIV-1-infected T cell lines generated VSV pseudotyped with functional HIV-1 Env that infect T cells via CD4 (right). CD4 antibodies and neutralising antibodies against Env prevent HIV-1 syncytium formation and pseudotype infection (bottom). (**b**) HIV-1 enters CD4^+^ T cells at neutral pH primarily via fusion with the T cell plasma membrane, leading to viral capsid entry into the cell cytoplasm and infection. “X” represents the yet-to-be-discovered coreceptors (CoR). (**c**) Soluble (and by extension membrane-anchored) forms of CD4 bind to the CD4 binding site on gp120 in the Env trimer (green circle) and induce conformation changes which prime Env for subsequent coreceptor binding and fusion. In T cell line-adapted strains of HIV-1, soluble CD4 triggers gp120 dissociation from gp41. Created in BioRender. Hofer, S. (2026) https://BioRender.com/q524537.

## Data Availability

No new data were created or analyzed in this study. Data sharing is not applicable to this article.

## References

[1] Dalgleish A.G., Beverley P.C., Clapham P.R., Crawford D.H., Greaves M.F., Weiss R.A. (1984). The CD4 (T4) antigen is an essential component of the receptor for the AIDS retrovirus. Nature.

[2] Klatzmann D., Champagne E., Chamaret S., Gruest J., Guetard D., Hercend T., Gluckman J.C., Montagnier L. (1984). T-lymphocyte T4 molecule behaves as the receptor for human retrovirus LAV. Nature.

[3] Sattentau Q.J., Weiss R.A. (1988). The CD4 antigen: Physiological ligand and HIV receptor. Cell.

[4] Sattentau Q.J., Clapham P.R., Weiss R.A., Beverley P.C., Montagnier L., Alhalabi M.F., Gluckmann J.C., Klatzmann D. (1988). The human and simian immunodeficiency viruses HIV-1, HIV-2 and SIV interact with similar epitopes on their cellular receptor, the CD4 molecule. AIDS.

[5] McClure M.O., Sattentau Q.J., Beverley P.C., Hearn J.P., Fitzgerald A.K., Zuckerman A.J., Weiss R.A. (1987). HIV infection of primate lymphocytes and conservation of the CD4 receptor. Nature.

[6] Maddon P.J., Dalgleish A.G., McDougal J.S., Clapham P.R., Weiss R.A., Axel R. (1986). The T4 gene encodes the AIDS virus receptor and is expressed in the immune system and the brain. Cell.

[7] Berger E.A., Murphy P.M., Farber J.M. (1999). Chemokine receptors as HIV-1 coreceptors: Roles in viral entry, tropism, and disease. Annu. Rev. Immunol..

[8] Sattentau Q.J., Dalgleish A.G., Weiss R.A., Beverley P.C. (1986). Epitopes of the CD4 antigen and HIV infection. Science.

[9] Arthos J., Deen K.C., Chaikin M.A., Fornwald J.A., Sathe G., Sattentau Q.J., Clapham P.R., Weiss R.A., McDougal J.S., Pietropaolo C. (1989). Identification of the residues in human CD4 critical for the binding of HIV. Cell.

[10] McClure M.O., Marsh M., Weiss R.A. (1988). Human immunodeficiency virus infection of CD4-bearing cells occurs by a pH-independent mechanism. EMBO J..

[11] Maddon P.J., McDougal J.S., Clapham P.R., Dalgleish A.G., Jamal S., Weiss R.A., Axel R. (1988). HIV infection does not require endocytosis of its receptor, CD4. Cell.

[12] Moore J.P., McKeating J.A., Weiss R.A., Sattentau Q.J. (1990). Dissociation of gp120 from HIV-1 virions induced by soluble CD4. Science.

[13] Moore J.P., McKeating J.A., Weiss R.A., Clapham P.R., Sattentau Q.J. (1991). Response: Receptor-Mediated Activation of Immunodeficiency Viruses in Viral Fusion. Science.

[14] Sattentau Q.J., Moore J.P. (1991). Conformational changes induced in the human immunodeficiency virus envelope glycoprotein by soluble CD4 binding. J. Exp. Med..

[15] Sattentau Q.J., Moore J.P. (1993). The role of CD4 in HIV binding and entry. Philos. Trans. R. Soc. Lond. B Biol. Sci..

[16] Weiss R.A., Clapham P.R., Cheingsong-Popov R., Dalgleish A.G., Carne C.A., Weller I.V., Tedder R.S. (1985). Neutralization of human T-lymphotropic virus type III by sera of AIDS and AIDS-risk patients. Nature.

[17] Klasse P.J., Sattentau Q.J., Moore J.P. (2026). How the HIV-CD4 interaction informs vaccine development. Viruses.

[18] Weiss R.A., Clapham P.R., Weber J.N., Dalgleish A.G., Lasky L.A., Berman P.W. (1986). Variable and conserved neutralization antigens of human immunodeficiency virus. Nature.

[19] Corti D., Langedijk J.P., Hinz A., Seaman M.S., Vanzetta F., Fernandez-Rodriguez B.M., Silacci C., Pinna D., Jarrossay D., Balla-Jhagjhoorsingh S. (2010). Analysis of memory B cell responses and isolation of novel monoclonal antibodies with neutralizing breadth from HIV-1-infected individuals. PLoS ONE.

[20] Zhou T., Lynch R.M., Chen L., Acharya P., Wu X., Doria-Rose N.A., Joyce M.G., Lingwood D., Soto C., Bailer R.T. (2015). Structural Repertoire of HIV-1-Neutralizing Antibodies Targeting the CD4 Supersite in 14 Donors. Cell.

[21] Burton D.R., Weiss R.A. (2010). AIDS/HIV. A boost for HIV vaccine design. Science.

[22] Steichen J.M., Madden P.J., Flynn C.T., Phulera S., Shil M., Kalyuzhniy O., Liguori A., Kifude C., Sewall L.M., Cottrell C.A. (2026). Vaccination elicits HIV broadly neutralizing antibodies in primates. Nature.

